# Emulgel Loaded with Flaxseed Extracts as New Therapeutic Approach in Wound Treatment

**DOI:** 10.3390/pharmaceutics13081107

**Published:** 2021-07-21

**Authors:** Cinzia Pagano, Claudio Baiocchi, Tommaso Beccari, Francesca Blasi, Lina Cossignani, Maria Rachele Ceccarini, Ciriana Orabona, Elena Orecchini, Enrico Di Raimo, Sara Primavilla, Laura Salvini, Alessandro Di Michele, Luana Perioli, Maurizio Ricci

**Affiliations:** 1 Department of Pharmaceutical Sciences, University of Perugia, 06123 Perugia, Italy; cinzia.pagano@unipg.it (C.P.); tommaso.beccari@unipg.it (T.B.); francesca.blasi@unipg.it (F.B.); lina.cossignani@unipg.it (L.C.); mariarachele.ceccarini@unipg.it (M.R.C.); maurizio.ricci@unipg.it (M.R.); 2 Department of Molecular Biotechnology and Health Sciences, Sect. Analytical Chemistry, Via Pietro Giuria 5, 10125 Torino, Italy; claudio.baiocchi@unito.it; 3 Department of Medicine and Surgery, University of Perugia, P.le Gambuli, 06132 Perugia, Italy; ciriana.orabona@unipg.it (C.O.); elena.orecchini@gmail.com (E.O.); 4 Istituto Zooprofilattico dell’Umbria e delle Marche, Via G. Salvemini 1, 06126 Perugia, Italy; e.diraimo@izsum.it (E.D.R.); s.primavilla@izsum.it (S.P.); 5 Fondazione Toscana Life Sciences, Via Fiorentina 1, 53100 Siena, Italy; l.salvini@toscanalifesciences.org; 6 Department of Physics and Geology, University of Perugia, 06123 Perugia, Italy; alessandro.dimichele@unipg.it

**Keywords:** flaxseed extract, chitosan, emulgel, antibacterial, anti-inflammatory, skin ulcers

## Abstract

Dry (D.E.) and liquid (L.E.) extracts were prepared from flaxseeds and their application in health field was evaluated. The chemical analysis showed that D.E. is rich in the lignan secoisolariciresinol diglucoside and L.E. in unsaturated triglycerides containing linolenic acid. Mainly, D.E. showed reducing (15.73 μmol Fe^2+^/g) and radical scavenging capacities (5.25 mg TE/g) and ability to down-regulate the expression of the pro-inflammatory cytokines NO (IC_50_ = 0.136 ± 0.009 mg/mL) and IL-6 (IC_50_ = 0.308 ± 0.103 mg/mL), suggesting its use in wound treatment. D.E. and L.E. were active against *S. pyogenes* and D.E. also against *S. aureus*. The two extracts were combined in a novel O/W emulgel in which the water phase was viscosized using a low molecular weight and highly deacetylated chitosan (1% wt./v). The presence of this polymer in the emulgel decreased the MIC values of the extracts. In fact, MIC shifted from 0.59 mg/mL to 0.052 mg/mL for D.E. and from 0.22 mg/mL to 0.036 mg/mL for L.E., concentrations safe both for keratinocytes and macrophages. Moreover, the emulgel demonstrated to inhibit *S. aureus*, *P. aeruginosa*, *S. pyogenes*, *E. coli,* and *K. pneumoniae* growth (inhibition halos 24–36 mm), strains often responsible for diabetic foot ulcer infection.

## 1. Introduction

**Figure 1:**
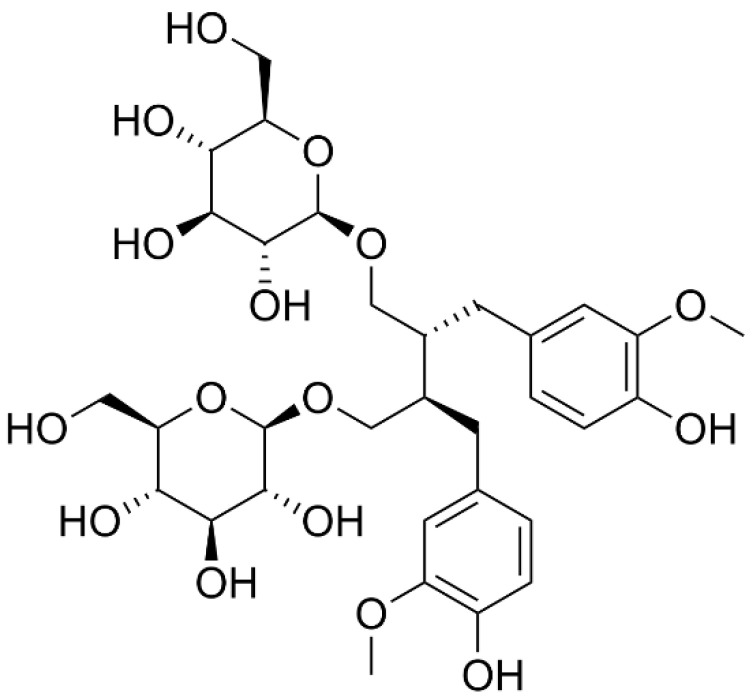
Chemical structure of secoisolariciresinol diglucoside (SDG).

Flax (*Linum usitatissimum*) is a plant belonging to *Linaceae* family cultivated since ancient times mainly for fiber (textile use) and oil production [1]. Flax plant produces pale blue flowers and fruit capsules containing small and brown seeds [2] rich in many inorganic and organic bioactive compounds. Among these (i) soluble fibers, also known as mucilage, including acidic compound of L-rhamnose (25.3%), L-galactose (11.7%), L-fructose (8.4%), D-xylose (29.1%), and other neutral polysaccharides as L-arabinose (20%) and D-xylose/D-galactose (76%) [3]; (ii) insoluble fibers, represented by cellulose (7–11%), lignin (2–7%) and acid detergent fibers (10–14%) [3]. Flaxseed coat is the richest source of the lignan phytoestrogen secoisolariciresinol diglucoside (SDG) (34–38%) (Figure 1) [2]; (iii) proteins, approximately 56−70%, found in cotyledons and about 30% in the coat and endosperm [3]; (iv) phenolic compounds such as gallic acid, protocatechuic acid, vanillic acid, caffeic acid, syringic acid, sinapic acid, p-coumaric acid, ferulic acid, and flavonoids [4].

Flaxseed oil is mostly found in cotyledons (75%) and less in seed coat and endosperm (22%) [3]. The main constituents of the oil are triglycerides of α-linolenic (52%), linoleic (17%), oleic (20%), palmitic (6%), and stearic (4%) acids [2].

The bioactive compounds contained in flaxseeds are responsible for many activities as anti-inflammatory (reducing the production of the pro-inflammatory cytokines IL-6 and TNF-α) and antiplatelet (inhibiting the anticoagulant prostacyclin) [2]. Soluble flaxseed fibers (mucilage) show antioxidant activity [5,6], regulate the gastrointestinal function, offer liver protection and reduce the risk of cardiovascular diseases [2]. There is considerable interest in the antioxidant potential of SDG due to the structural similarity to the aglycone secoisolariciresinol (SECO) and to nordihydroguaiaretic acid (NDGA).

Flaxseeds show antimicrobial activity attributable both to lignans and phenolic acids [7] as well as to long-chain unsaturated fatty acids, especially α-linolenic and linoleic acids [8]. Flaxseeds are largely used as a food supplement for cholesterol levels control or for gastrointestinal function regularization. Flaxseed oil is also largely used in commercially available cosmetic products for the anti-wrinkle activity, skin soothing and nourishing, repairing effect and hair growth stimulation. Flaxseeds, mainly as flour, find interesting applications for the preparation of functional foods [9]. It has become an attractive ingredient in the diet specially planned for specific health benefits, for example flaxseeds represent a rich source of omega 3. Despite the numerous flaxseed properties, its use in health products as medical devices and medicines is very limited [10].

The aim of this work was to prepare extracts from flaxseeds and to characterize and formulate them in a suitable formulation. The research was divided in three steps (i) preparation of two flaxseed extracts, dry and liquid; (ii) characterization of the extracts and evaluation of their activities (antioxidant, anti-inflammatory, antibacterial); (iii) development and characterization of a suitable formulation on the basis of the observed activities.

The properties of the prepared extracts showed their applicability in the treatment of wounds. For this reason, a semisolid formulation was developed, useful for self-administration and adaptable to every surface. An O/W emulgel was prepared, introducing the dry extract in the external water phase and the liquid extract, due to the lipophilicity, in the internal oil phase. The W phase was viscosized with chitosan characterized by low molecular weight and high deacetylation degree. The use of this excipient allowed to obtain a formulation with marked antimicrobial activity against *S. aureus*, *P. aeruginosa*, *S. pyogenes*, *E. coli* and *K. pneumoniae* strains often responsible for diabetic foot ulcers infection. Moreover, the combination of the two extracts to chitosan considerably decreased the MIC values of the two pure extracts, suggesting a synergistic effect.

## 2. Materials and Methods

### 2.1. Materials

#### 2.1.1. Chemical Part

Flaxseed flour and refined linseed oil were purchased from A.C.E.F. (Fiorenzuola D’Arda, Italy). *Linum usitatissimum* seeds p.e. 20% lignans (produced by Fontana) was purchase from Farmalabor (Canosa di Puglia, BT). Absolute ethanol (EtOH abs), ethanol 96% (EtOH), Folin–Ciocalteu reagent, TPTZ (≥98%), Trolox (97%), ABTS (≥98%), HCl, FeCl_3_, NaOAc, Na_2_CO_3_, AcOH, and gallic acid were purchased from Sigma–Aldrich (Milano, Italy).

Chitosan FG90 (deacetylation degree 99.97%, MW 100 KDa, viscosity of 1% wt. solution in 1% acetic acid 110 mPa.s) was produced and characterized by Prof. Riccardo Muzzarelli, Department of Biochemistry, Biology and Genetics–Università Politecnica delle Marche-Ancona, Italy. Vaseline, liquid paraffin, cetomacrogol 1000 and cetostearyl alcohol were purchased from Galeno (Carmignano, Italy). MilliQ system Millipore (Rome, Italy) was used to produce ultrapure water.

#### 2.1.2. Microbiological Part

Test media were prepared as follow:

- Agar-well diffusion test medium; deionized water (containing agar 13%), meat extract (3%), sodium chloride (10%), glucose (4%), dibasic potassium phosphate (1%) and meat peptone (5%); after preparation, the test medium was autoclaved.
- Brain Heart Infusion (BHI) Broth; deionized water, BHI (3.7%, Biolife Italiana Srl, Milano, Italy).
- Mueller Hinton Broth with 5% Blood; deionized water, Mueller Hinton Broth (2.2%, Biolife Italiana Srl, Italy), Horse Lysate Blood (5%, Allevamenti Blood di Fiastra Maddalena).
- 5% Sheep Blood Agar; deionized water, Columbia Agar Base (4.4%, Microbiol Srl, Macchiareddu, Cagliari, Italy), Defibrinated Sheep Blood (5%, Allevamenti Blood di Fiastra Maddalena). Bacterial suspension at concentrations of 1 × 10^5^ CFU/mL was used for the antimicrobial test.

#### 2.1.3. Biochemical Part

DMSO was purchased from Thermo Fisher Scientific (Waltham, MA, USA). MTT was purchased from Sigma–Aldrich Srl (St. Louis, MO, USA). Trypsin (EDTA), streptomycin, penicillin, glutamine, Fetal Bovin Serum (FBS), Dulbecco’s modified Eagle medium (DMEM), PBS (Phosphate buffered saline) were purchased from Microtech srl (Pozzuoli, NA, Italy). CytoSelect 24-Well Wound Healing Assay was supplied by CELL BIOLABS, INC. (San Diego, CA, USA).

### 2.2. Methods

#### 2.2.1. Extraction Procedure

The dry extract (D.E.) was prepared by a method already used with success for other vegetal matrices [11,12] and properly adapted for this matrix. Flaxseed flour (2 g) was suspended in 80 mL of a hydroalcoholic solution (EtOH/water 60:40 *v*/*v*) and kept at 45 °C under magnetic stirring (800 rpm) for 90 min, the obtained suspension was then centrifuged (4000 rpm, 10 min, R.T.). The supernatant was recovered by filtration (cellulose membrane filter—Whatman 41, Whatman GmbH, Dassel, Germany). The solvent was removed by rotary evaporation at 35 °C. The solid was then solubilized in 10 mL of bidistilled water and freeze-dried (Hetodrywinner, Analytical Control De Mori, Milano, Italy). The liquid extract (L.E.) was prepared as follows: flaxseed flour (100 g) was wetted and macerated with 200 mL of EtOH in a percolator and kept in these static conditions at R.T. for 90 min. After this time, the percolate was collected, and the solvent removed by a rotary evaporator at 35 °C.

### 2.3. Extracts Characterization (D.E. and L.E.)

#### 2.3.1. Chemical Analysis

D.E. (500 mg) was suspended in 10 mL of water/MeOH 40:60 *v*/*v*, sonicated for 30 min and then centrifuged (10,000 rpm, 15 min, R.T.). Twenty µL of supernatant were injected in an Ultimate 3000SD UPLC system (ThermoFisher Scientific, Bremen, Germany) coupled via an Electrospray source to a Q-Exactive Plus (ThermoFisher Scientific, Bremen, Germany). Chromatographic conditions. Acquity Waters CSH C18 column, 2.1 × 150 mm, d_p_ 1.7 µm, thermostated at 35 °C. Eluent A: aqueous formic acid (0.1%), eluent B: acetonitrile +0.1% formic acid. Gradient conditions: 0–1 min 2% B, 1–40 min from 2 to 90% B, isocratic step at 90% of B for 5 min. Flow rate 0.250 mL/min. Mass spectrometry conditions. Multistage HR-MS spectra were recorded in ion-negative mode, spray voltage 3.1 kV, sheath gas 20 (arbitrary units), auxiliary gas 5.0 (arbitrary units), capillary temperature 320 °C and resolution 17,500. MS/MS spectra were obtained by a Higher Energy Collision Dissociation (HCD) of 35 (arbitrary units).

L.E. (100 µL) was diluted in 1 mL of EtOH:MeOH:iso-propanol: ammonium acetate 20 mM (75:17:7:1) and analyzed by direct infusion in Q-Exactive Plus ESI source at a flow rate of 5.0 µL/min. Spectra were recorded in ion positive mode (Resolution 70,000) in a *m*/*z* values range 100–2000. Spray voltage 3.5 kV, sheath gas (N_2_) 10 (arbitrary units), capillary temperature 250 °C, R-Lens 200 volts. MS/MS spectra were obtained by a Higher Energy Collision Dissociation (HCD) ranging from 15 to 35 (arbitrary units) depending on the species selected.

#### 2.3.2. Total Phenol Content and Antioxidant Activity

D.E. and L.E. antioxidant activity was evaluated by the measurement of the total phenolic content (TPC), ferric reducing antioxidant power (FRAP) and 2,2′-azino-bis (3-ethylbenzothiazoline-6-sulphonic acid) (ABTS).

The TPC was measured by Folin–Ciocalteu assay [13]. D.E. or L.E. (25 mg/mL in EtOH) was diluted with water (1:10) and then 1 mL of this solution mixed with of Folin–Ciocalteu reagent (0.5 mL), 20% Na_2_CO_3_ solution (2.0 mL) and water (6.5 mL) and kept 90 min at R.T. protected from light. The sample was analyzed spectrophotometrically (ʎ_max_ = 750 nm) using a calibration curve of gallic acid, and the results expressed as mg of gallic acid equivalents (GAE) per gram of dry flaxseed flour (mg GAE/g), *n* = 2. D.E. and L.E. reducing power was measured by FRAP assay [14]. D.E. or L.E. aqueous solution (100 μL having a concentration of 2.5 mg/mL) were diluted with bidistilled water (900 μL) and added by FRAP reagent (2 mL). The sample was maintained for 30 min protected from light and then analyzed (ʎ_max_ = 593 nm) using FeSO_4_ as standard solutions for the preparation of the calibration curve. The final values were expressed as μmol Fe^2+^ per gram of dry flaxseed flour (μmol Fe^2+^/g), *n* = 2.

D.E. and L.E. radical scavenging activity was measured by ABTS assay [15]. ABTS radical cation (ABTS^•+^) was produced by mixing ABTS solution and K_2_S_2_O_8_ [15]. ABTS aqueous solution (7 mmol/L) was prepared and K_2_S_2_O_8_ (3.31 mg) was added to 5 mL of this solution. The sample was kept overnight protected from light at R.T. overnight. After this period, it was diluted with bidistilled water in order to produce an absorbance of ~0.70 at ʎ_max_ = 734 nm. Flaxseed flour aqueous solution (60 μL having a concentration 1.25 mg/mL) was added by diluted ABTS^•+^ (4 mL) and left protected from light for 6 min then spectrophotometrically analyzed (ʎ_max_ = 734 nm) using a calibration curve of Trolox (from 0.1 to 0.5 mg/mL). The antioxidant capacity was expressed as mg Trolox equivalents (TE) per gram of dry flaxseed flour (mg TE/g), *n* = 2.

#### 2.3.3. Antimicrobial Activity Assay

The antimicrobial activity of the samples was evaluated against the strains reported in Table 1. The stored strains were revitalized on Brain Heart Infusion Broth and incubated according to the growth conditions shown in Table 1.

The experiments were performed using the agar-well diffusion technique, properly modified [16], using a test medium pH 7.2 (see recipe Section 2.1). The bacterial suspension (1 mL) was added to the medium after cooling (at 45–48 °C) reaching a concentration of 1 × 10^5^ CFU/mL. Different inoculated media were used for each bacterial strain. The suspensions were mixed and poured (20 mL) into Petri dishes (90 mm diameter), then cooled on a horizontal surface. The sample (100 µL) was punt in a hole of 13 mm in diameter in the center of agar then incubated according to the specific growth conditions (Table 1), *n* = 3 for each strain. Three inoculated agar plates were incubated to assess medium sterility. After the incubation time, the inhibition halo was measured by a gauge.

The minimum inhibitory (MIC) and minimum bactericidal (MBC) concentrations were determined for *S. pyogenes*, by a standard microdilution technique, according to Clinical Laboratory Standards Institute guidelines. The bacterial suspension used for the assay was prepared, adjusting the number of bacteria to approximately 1 × 10^5^ CFU/mL with fresh Mueller Hinton Broth with 5% Blood. Moreover, three controls were set up: these included antibiotic control (with ciprofloxacin), organism control (wells containing Mueller Hinton Broth with 5% Blood and the bacterial suspension), negative control (wells containing Mueller Hinton Broth with 5% Blood and the extract at the same concentration tested). The microplate was incubated for 24 h at 37.0 °C ± 1.0 in aerobic conditions. MIC was defined as the lowest concentration of extract that produced no bacterial growth when compared to time 0 wells. Moreover, in order to define the MBC, 100 µL of each well were plated on 5% Sheep Blood Agar dishes, then incubated for 24 h at 37.0 °C ± 1.0 in aerobic conditions. The MBC was represented as the smallest amount of extract that was capable of killing the microbial inoculum, demonstrated by the total absence of growth.

#### 2.3.4. Cell Culture and Viability

Two different cell lines, RAW 264.7 and HaCaT, were used to investigate, respectively, anti-inflammatory activity and epidermal homeostasis after D.E. treatment. The first one is a mouse macrophage purchased from the American Type Culture Collection (ATCC, Manassas, VA, USA), whereas the second one is a human immortalized keratinocyte obtained from I.Z.S.L.E.R. Cell lines were cultured according to standard procedures: RAW 264.7 growth in Roswell Park Memorial Institute 1640 medium (RPMI-1640) and HaCaT in Dulbecco’s modified Eagle’s medium (DMEM). 10% heat-inactivated Fetal Bovine Serum (FBS),100 U/mL penicillin, 100 µg/mL streptomycin and 2 mM of L-glutamine were used as a supplement for both media. RAW 264.7 and HaCaT were tested for mycoplasma contamination before use. MTT (3,(4,5-dimethylthiazol-2)2,5 difeniltetrazolium bromide) assay was used to test cellular viability after treatment for 24 h with different concentration of D.E. [17]. As previously described, 1 × 10^4^ cells were seeded in a 96 well plate and after 24 h scalar concentrations of D.E. were used for treatment. 0.5 μg/μL of MTT reagent was added to the culture and after 3 h the supernatant was carefully removed. 100 µL DMSO was added to each well to dissolve formazan salt crystals and after 30 min the absorbance (OD) values were measured spectrophotometrically at 540.0 nm. The experiments were repeated two times in triplicate for the D.E. extract, whereas the same tests were not performed on L.E. as, due to the lipophilicity (oil), the mixing with the culture media was not possible (emulsion formations).

Each experiment was performed two times in triplicate. Cell viability was expressed as a percentage relative to that of the control cells as described previously [17,18].

#### 2.3.5. Anti-Inflammatory Activity

RAW 264.7 cells were activated by the stimulation with 50 ng/mL of lipopolysaccharides (LPS), serotype 055:B5 (Sigma–Aldrich, Saint Louis, MO, USA), for 24 h and the anti-inflammatory activity investigated according to the procedures described in a previous work [11].

### 2.4. Emulgel Preparation and Characterization

The emulgel having the composition

- oil phase (O): L.E. 22.0 g, cetostearyl alcohol 6.0 g, cetomacrogol 1000 2.0 g
- water phase (W): FG90 1% wt. solution 69.0 g, D.E. 1.0 g,

was prepared as follows. The oil phase (O) represented by cetomacrogol 1000 and cetostearyl alcohol were melted in a steam bath, then L.E. was added to the melted mass. The gelled water external phase (W), represented by D.E. solubilized in FG90 1% (wt./v) solution (acetic acid 1% *v*/*v*), after warming, was added to the oil phase (O) and stirred (600 rpm) until complete cooling.

#### 2.4.1. In Vivo Evaluation of the Formulation Skin-Feel

The skin-feel of the prepared emulgel was assayed by three healthy volunteers who were asked to apply the formulation to the skin and make a judgment about (i) sensation during the application and massage (pleasant or unpleasant), (ii) physical appearance and (iii) greasiness degree.

#### 2.4.2. Viscosity Measurement

The viscosity of the prepared emulgel was measured by a Stresstech HR (Reologica Instruments, AB Milan, Italy) rheometer, (cone-plate geometry, diameter 40 mm, angle 1°). The shear stress was set in the range 1–100 Pa working at 32.0 °C ± 0.1 (skin surface temperature), n = 3 ± SD.

#### 2.4.3. Scanning Electron Microscopy

The dimensions of the inner oil phase of the emulgel were evaluated by Field Emission Scanning Electron Microscopy (FE-SEM LEO 1525 ZEISS) using an electron high tension of 5 and 15 kV. The sample was spread on stubs with double sided adhesive carbon tape and metalized with chromium (8 nm). The images were obtained using secondary electron (SE) and In-lens detectors at magnifications of 1.00 KX. The average size distribution of droplets was determined by ImageJ software using SEM images.

## 3. Results

### 3.1. Dry and Liquid Extracts Preparation

**Figure 2:**
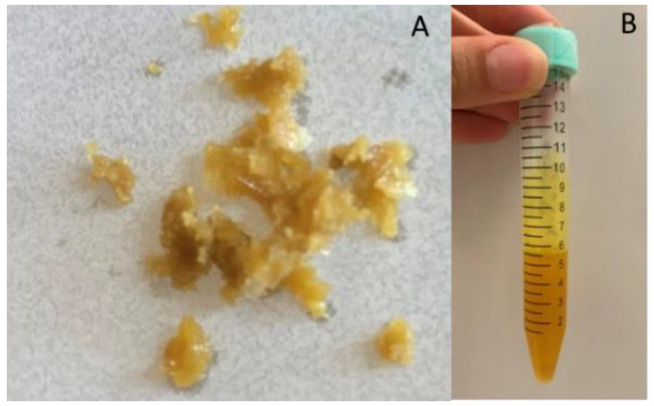
Pictures of dry extract (D.E.) (**A**) and liquid extract (L.E.) (**B**).

The dry extract (hereafter cited as D.E.), obtained by freeze-drying procedure described in method section (Section 2.2.1), appears as yellow dry powder (Figure 2A) having a water solubility of 5 mg/mL at 25 °C. The solution shows a light opalescence due to mucilage presence in the extract. Until use, D.E. was stored under P_2_O_5_ and protected from light in order to avoid degradation processes due to both humidity and UV rays. The extraction method used and the lyophilization procedure allowed to obtain an overall yield of 12.5% ± 0.2, calculated from the amount of flaxseed flour used as starting material for the extraction procedure. Liquid extract (hereafter cited as L.E.), obtained by the procedure described in method Section 2.4, appears as an orange-yellow solution with high density (Figure 2B). Until use it was stored at 4–5 °C. The extraction method used allowed one to obtain an overall yield of 14.2% ± 0.01.

### 3.2. Extracts Characterization

#### 3.2.1. Chemical Analysis

Extracts qualitative composition was determined by HPLC-High Resolution Mass Spectrometry. At this purpose, the distribution of unsaturated triglycerides (TAGs) containing linolenic acid was detailed in the case of L.E., whereas in the case of D.E., the presence of the flaxseed typical lignan SDG (Secoisolariciresinol Di-Gliceride) was confirmed as well.

**Figure 3:**
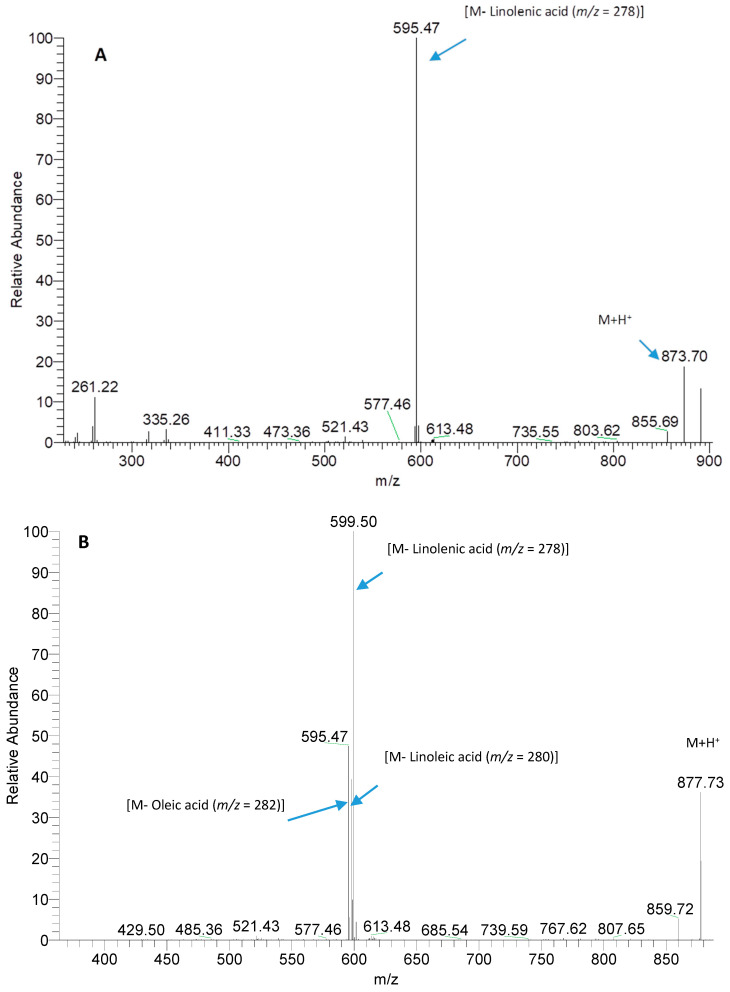
Mass spectrum of triglyceride (TAG) LnLnLn (**A**) and of TAG LnLO (**B**). Ln = Linolenic acid, L = Linoleic Acid, O = Oleic acid.

The presence of TAG molecules in L.E. was evaluated by their mass spectra during direct infusion in the mass spectrometer. As example, in Figure 3A,B, the typical high resolution MS/MS spectrum of both TAG compositionally symmetric (LnLnLn) and TAG totally asymmetric (LnLO) are reported.

TAGs typically fragment, losing the fatty acids located in position 1 and 3 of glycerol. In mass spectrum reported in Figure 3A TAG is compositionally symmetric so just one product ion (*m*/*z* value 595.47) is detectable, corresponding to the loss of linolenic acid located in all glycerol positions. In mass spectrum reported in Figure 3B three product ions are detectable (*m*/*z* values 597.50, 595.47, 593.49 in order of intensity) traceable back to the loss of fatty acids Ln, O, L, respectively. This behavior, together with the obtained accurate molecular weight of the whole molecule, allowed to qualitative characterize TAG content.

The more present TAGs identified were: LnLnLn, LnLO, LnLnL, PLnLn, LPLn, and OPLn in order of concentration (P = Palmitic acid). As expected, linolenic acid is a component of the major part of the triglycerides identified.

**Figure 4:**
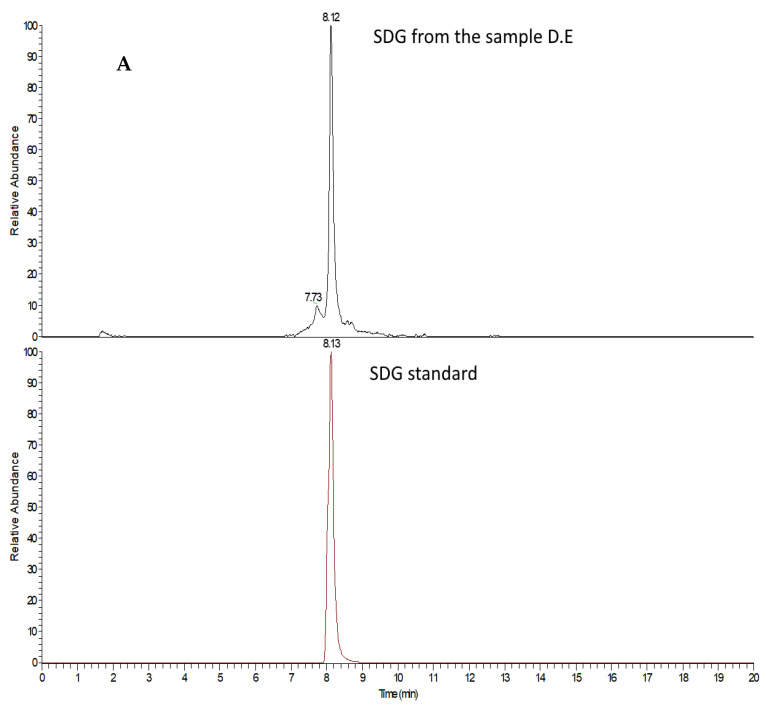

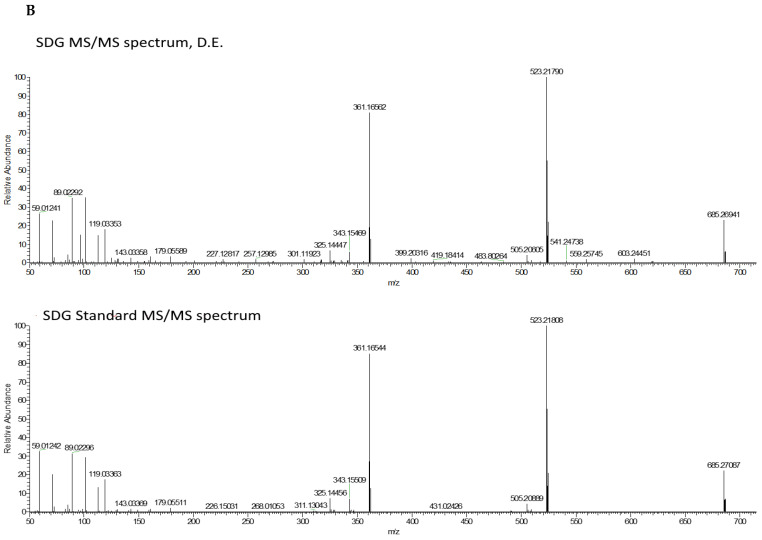
Comparison of retention time of SDG in D.E. and of SDG pure standard (**A**). Comparison of MS/MS spectra of SDG in dry extract and of SDG pure standard (**B**).

The identification of SDG molecule in D.E. was based on the retention time coincidence of both SDG contained D.E. and that of pure standard SDG together with the overlapping of their MS/MS high resolution mass spectra showing a double loss of two glucose molecules (Figure 4A,B).

#### 3.2.2. Total Phenolic Content and Antioxidant Activity

Folin–Ciocalteu method was used to measure TPC of both D.E. and L.E. samples. The obtained results (Table 2), expressed as mg GAE/g dry flaxseed flour, showed values of phenolic content of 1.94 mg GAE/g and 1.62 mg GAE/g for D.E. and L.E., respectively. These values are comparable to the results obtained from Teh et al. [19], which studied the effect of different solvent systems for phenol extraction from defatted oilseed cake. In another paper, Deng et al. measured both the antioxidant activity and the bioactive compounds of Chinese flaxseed according to variety and geographical origin and reported TPC values ranging from 109.93 (Ningxia) to 246.88 mg GAE/100 g (Inner Mongolia) [20].

The reducing capacity of the flaxseed extracts was evaluated by FRAP assay and the results expressed as μmol Fe^2+^ per gram of dry flaxseed flour (μmol Fe^2+^/g) (Table 2). The results showed that both samples contained substances having reducing capacity (electron donating), in fact, values of 15.73 μmol Fe^2+^/g and 11.69 µmol Fe^2+^/g for D.E. and L.E., respectively, were obtained. These values are higher compared to the results reported by other authors [7], which found 0.51 μmol Fe^2+^/g fresh flaxseed cake for ethanolic extract and 0.93 μmol Fe^2+^/g for hydroalcoholic extract obtained with 80% methanol. The results of ABTS assay showed that D.E. sample had higher antiradical activity towards ABTS^•+^ in comparison to L.E. sample (5.25 vs. 0.62 mg TE/g dry flaxseed flour). D.E. ABTS value was in accordance with the results obtained by Tawaka et al. [21] for *Linum pubescens* Banks & Sol., which ranged from 12.9 μmol TE/g (aqueous extract) to 37.6 μmol TE/g (methanolic extract).

#### 3.2.3. Antimicrobial Activity

**Figure 5:**
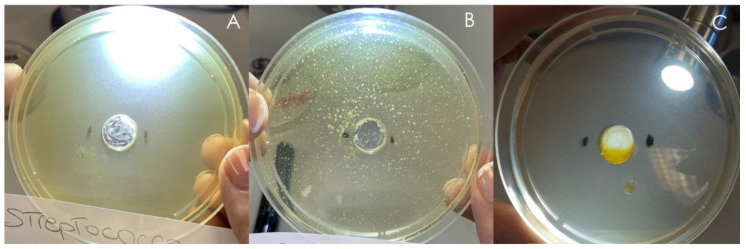
Inhibition halos measured for D.E. (**A**) *S. pyogenes* (**B**) *S. aureus*; for L.E. *S. pyogenes* (**C**).

D.E. and L.E. antimicrobial activity was studied by the modified agar diffusion method [16,22]. A preliminary characterization was performed on the bacterial strains and yeast reported in Table 1. Two different D.E. concentrations were assayed, 100 mg/mL and 150 mg/mL. In order to perform a comparison, also a marketed D.E. (tit. 20% lignans) was included in the experiment at the same concentrations. In the case of L.E., it was assayed as it is (no dilutions) and compared to a commercial flaxseed oil. D.E. was active against the Gram+ bacteria *S. pyogenes* measuring an inhibition halo of 20 mm both at 100 mg/mL and 150 mg/mL (Table 3, Figure 5). The concentration 150 mg/mL also produces the inhibition of the Gram+ bacteria *S. aureus* growth (Table 3) while no activity was observed against the tested Gram− and yeast. Marketed D.E. was active against the Gram+ bacteria *S. pyogenes*, *S. epidermidis* and *S. aureus* (Table 3). L.E. was active against the Gram+ *S. pyogenes* (Figure 5) while no activity was observed against the tested Gram− and yeast; the marketed oil did not show activity. From the data observation it is possible hypothesize that the antimicrobial activity is attributable to the presence of lignans and phenolic acids [8] or to the presence of long-chain unsaturated fatty acids like α-linolenic acid and linoleic acid [7]. 

#### 3.2.4. Cytotoxic and Anti-Inflammatory Activity of D.E. in LPS-Stimulated RAW 264.7 Cell Line

**Figure 6:**
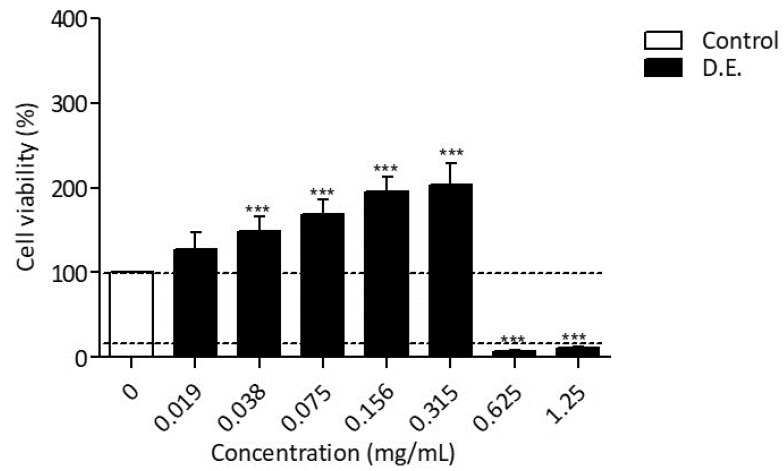
MTT assay on RAW 264.7 cells treated with different D.E. concentrations and incubated for 24 h. RAW 264.7 cells co-treated with lipopolysaccharide (LPS) was used as Control, *n* = 6 ± SD. Dotted lines indicate 100% and 6%, respectively. *** *p* < 0.0001, D.E. vs. Control (One-way ANOVA test).

D.E. anti-inflammatory activity was studied by the murine macrophage cell line RAW 264.7 stimulated with lipopolysaccharide (LPS, 50 ng/mL for 24 h). In order to exclude false positives, D.E. cytotoxicity on RAW 264.7 cell line was investigated as well. By using ten two-fold dilutions of D.E in the concentration range 0.019–1.25 mg/mL, it was observed that cell viability reached values below 6% at concentrations >0.315 mg/mL after 24 h of incubation. In the concentration range of 0.019–0.315 mg/mL cell viability was maintained higher than 100%, suggesting a proliferative effect of D.E. on RAW 264.7 cells (Figure 6).

**Figure 7:**
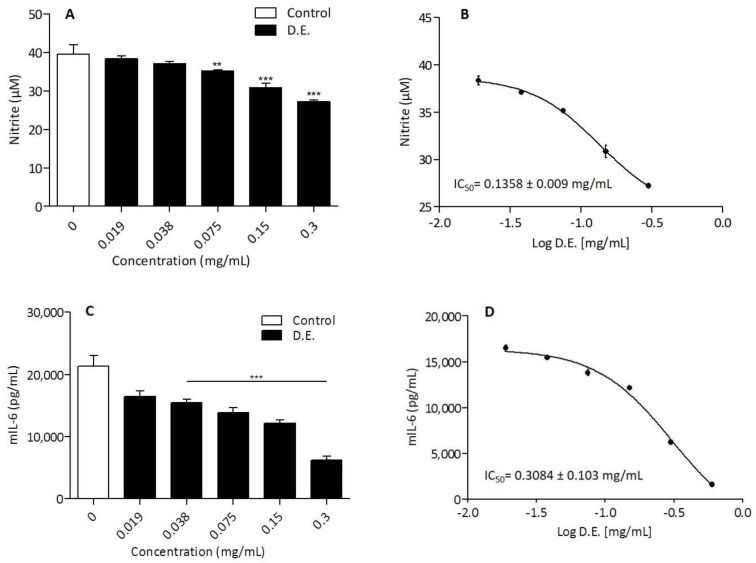
(**A**) RAW 264.7 cells were in vitro co-treated with LPS (Control) and different concentrations of D.E. for 24 h. NO concentration in the culture supernatants was quantified by using Griess reagent. Results are reported as mean ± SD of 3 independent experiments, each conducted in triplicate. ** *p* < 0.001, *** *p* < 0.0001, D.E. vs. Control (One-way ANOVA test). (**B**) Concentration–response curve was obtained for the determination of the IC_50_. Results are reported as mean of 2 independent experiments, each conducted in triplicate. The IC_50_ value, equivalent to the sample concentration that inhibits NO production by 50%, was determined using non-linear regression analysis. (**C**) RAW 264.7 cells were in vitro co-treated with LPS (Control) and different concentrations of extract (D.E.) for 24 h. IL-6 concentration in the culture supernatants was determined by ELISA test. *** *p* < 0.0001, D.E. vs. Control (One-way ANOVA test). (**D**) Concentration–response curve was obtained for the determination of the IC_50_. Results are reported as the mean ± SD of three independent experiments, each conducted in triplicate. The IC_50_ value, equivalent to the sample concentration that inhibits cytokine production by 50%, was determined using non-linear regression analysis.

Based on the cytotoxicity results, the anti-inflammatory effect of D.E. in LPS-treated RAW 264.7 cell line was investigated in the concentrations range of 0.019–0.315 mg/mL. The incubation of LPS-stimulated RAW 264.7 with D.E. for 24 h induced a significant decrease of NO release in the concentration range of 0.075–0.300 mg/mL (Figure 7A). The obtained concentration curve provided an IC_50_ = 0.136 ± 0.009 mg/mL for the downregulation of NO release by D.E. (Figure 7B).

The release of pro-inflammatory cytokines, IL-6, TNF-α and IL-1β in the same cell system was analyzed as reported in the literature [11]. Specifically, IL-6 secretion by LPS-treated RAW 264.7 macrophages was significantly inhibited by D.E. starting from the concentration of 0.038 mg/mL (Figure 7C). The inhibitory effect on IL-6 production was concentration-dependent with an IC50 = 0.308 ± 0.103 mg/mL (Figure 7D). Differently from IL-6 inhibition, the inhibitory effect of D.E. on IL-1β and TNF-α did not show a concentration dependence.

**Figure 8:**
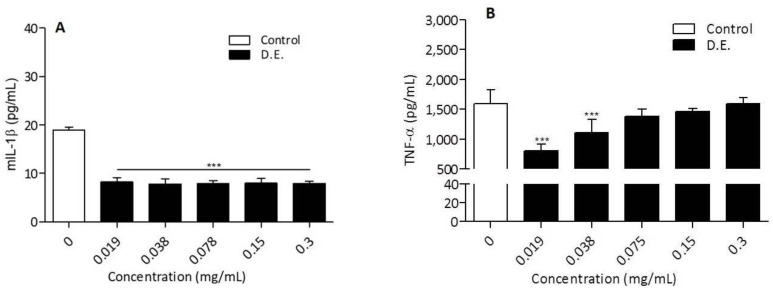
RAW 264.7 cells were in vitro co-treated with LPS (Control) and different concentrations of extract D.E. for 24 h. IL-1β (**A**) and TNF-α (**B**) concentrations in the culture supernatants were determined by ELISA test. *** *p* < 0.0001, D.E. vs. Control (One-way ANOVA test). Results are reported as the mean of 2 independent experiments, each conducted in triplicate.

For the first, all the tested concentrations significantly inhibited IL-1β secretion independently from D.E. concentration (Figure 8A). In the case of TNF-α production, a significant inhibition was observed at the lowest D.E. concentrations (0.019 and 0.038 mg/mL), while no inhibition was observed at higher concentrations (0.075–0.300 mg/mL) (Figure 8B). Although the secretion of both IL-1β and TNF-α is inhibited by D.E. in dose-independent manner, both cytokines were inhibited by D.E. at concentrations safe for cells, below the cytotoxic concentration (i.e., 0.6 mg/mL).

**Figure 9:**
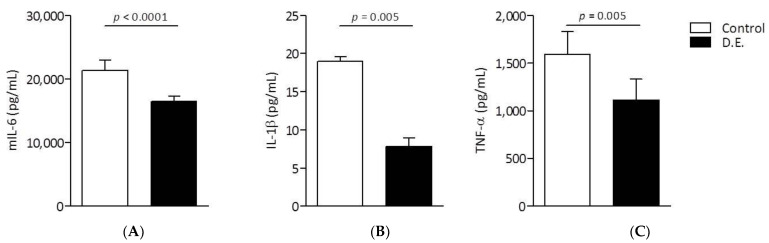
RAW 264.7 cells were in vitro co-treated with LPS (Control) and D.E. at 0.038 mg/mL for 24 h. Supernatants were collected and the concentrations of IL-6 (**A**), IL-1β (**B**) and TNFα (**C**) were determined by ELISA test. D.E. vs. Control (unpaired Student’s *t*-test).

Overall, D.E. can exert an anti-inflammatory activity in a variable concentration range below the cytotoxic concentrations (Figure 9).

#### 3.2.5. In Vitro Cytotoxic Effect on Keratinocytes

**Figure 10:**
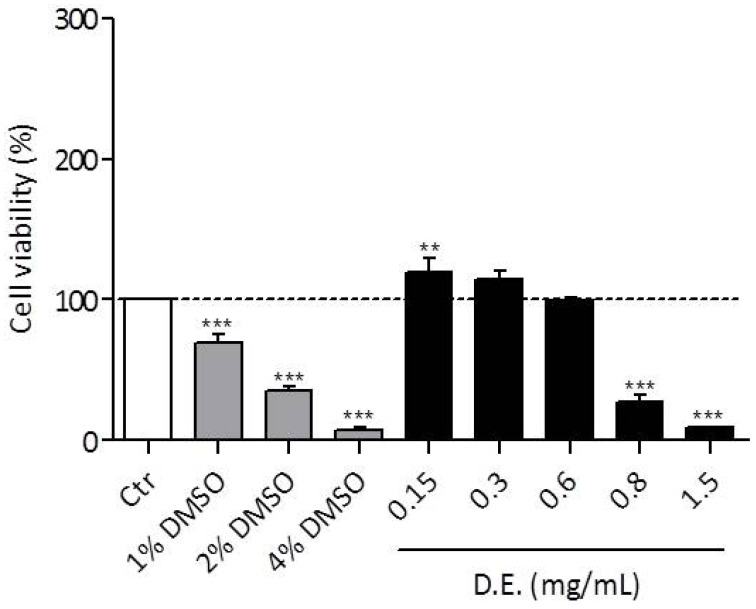
Viability measured in vitro on HaCaT cells for different D.E. concentrations. Ctr, untreated cells in DMEM was set at 100%, three different percentages (1%, 2% and 4%) of DMSO were used as positive controls. ** *p* < 0.001, *** *p* < 0.0001, D.E. vs. Ctr (One-way ANOVA test).

The performed characterization suggested that the prepared extracts could find interesting applications for the treatment of wounds in which the antioxidant, anti-inflammatory and antimicrobial activities are useful to enhance the repair process. With this idea, it was considered useful to evaluate in vitro D.E. safety by MTT test (cytotoxicity study). With this purpose, human keratinocytes (HaCaT) were chosen as cell line representative of stratum corneum. The obtained results showed that D.E. is safe in the concentration range of 0.15–0.6 mg/mL as the viability of the tested cells was maintained ≥90.00% (Figure 10). Increasing the concentration from 0.8 to 2.5 mg/mL, cell viability decreased under the acceptability value (<60%).

The experiments were not performed on L.E. as, due to the lipophilicity (oil), the mixing with the culture media was not possible (emulsion formations).

### 3.3. Formulation of D.E. and L.E.

The next step of this study was the choice of the most appropriate formulation suitable for D.E. and L.E. topical application. The preliminary characterization of these two extracts showed that they possess suitable properties as antioxidant, antimicrobial as well as anti-inflammatory activities that make them interesting to be used as active ingredients in products intended for wound treatment. With the idea to combine both D.E. and L.E. in the same formulation, a biphasic system represented by an oil in water (O/W) cream was planned and developed. In fact, D.E., due to the hydrophilic properties, was introduced in the external water phase while L.E., due to its lipophilic nature, was introduced in the internal oil phase.

With the aim to find the most suitable composition, the recipe of the hydrophilic base cream (O/W emulsion), reported in the Farmacopea Ufficiale Italiana (F.U. XII Ed.) “cetomacrogol base cream” [23] was used as starting composition. The recipe described in the monograph, based on 30/70 ratio of O/W, is the following:

- Oil phase (O): vaseline 15 g, liquid paraffin 6 g, cetostearyl alcohol 7.2 g, cetomacrogol 1.8 g;
- Water phase (W): water 70 g.

The first modification of the original recipe consisted in the replacement of both vaseline and liquid paraffin (O phase) with L.E (1% wt.) while D.E. was solubilized in the external water phase. The obtained cream showed consistency problems and was greasy and unpleasant. After further modification attempts, the most suitable composition was the following:

- Oil phase (O): L.E. 22 g, cetostearyl alcohol 6.0 g, cetomacrogol 2.0 g.
- Water phase (W): water 69 g, D.E. 1.0 g.

Then, in order to obtain a more pleasant and stable formulation, the water phase was replaced by a hydrogel, in which the oil phase droplets are dispersed in a polymeric network that prevents the possible coalescence. Thank to this modification an emulgel with excellent consistency and appearance was prepared.

In the choice of the most suitable polymer to use for the W phase gelification, it was taken into account an important aspect of the antimicrobial activity results obtained from the raw D.E. and L.E. In fact, as shown in Table 3, D.E. is active against *S. pyogenes* and *S. aureus* using the concentration of 150 mg/mL, cytotoxic for both macrophages (Figure 6) and keratinocytes (Figure 10). The concentration range useful to obtain the anti-inflammatory activity is definitely lower than 150 mg/mL (0.019–0.300 mg/mL). Thus, the problem was: how maintain both antimicrobial and anti-inflammatory activities preserving cells safety? Some authors described the ability of the polymer chitosan (with low molecular weight and high deacetylation degree), combined to antimicrobial agents, to enhance the activity, decreasing their MIC value [24,25]. Considering this aspect, chitosan was chosen for the external water phase gelification. Chitosan is a biocompatible and non-toxic polymer and thus useful for this application [26]. For this formulation FG90 chitosan, having a molecular weight of 100 kDa and deacetylation degree of 99.97%, was chosen [27]. The choice of this type of chitosan is also due to the documented antimicrobial activity [28,29,30] attributable to its polycationic character that favors the interaction with cell walls and cytoplasmic membranes negatively charged. These interactions result in decreased osmotic stability, membrane disruption and eventual leakage of intracellular elements [31].

**Figure 11:**
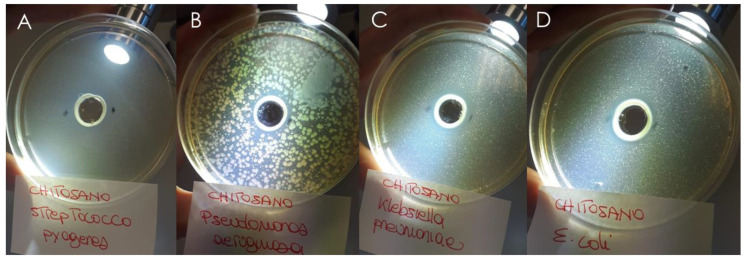
Inhibition halos measured for FG90 1% wt. solution for (**A**) *S. pyogenes*, (**B**) *P. aeruginosa*, (**C**) *K. pneumoniae* and (**D**) *E. coli*.

Thus, a hydrogel containing 1% wt. FG90 chitosan was prepared and used to replace the water phase of the optimized recipe. Before its preparation, FG90 hydrogel 1% wt. (obtained using 1% acetic acid–water solution) was submitted to preliminary in vitro antibacterial studies in order to know its specific activity. The experiments were performed using the diffusion method adopted for both D.E. and L.E. (Section 2.3) as well as the same strains. The obtained results showed that FG90 is active against the Gram+ *S. pyogenes* (inhibition halo measured 25 mm) and against the Gram− bacteria *P. aeruginosa* (inhibition halo measured 25 mm), *K. pneumoniae* (inhibition halo measured 23 mm) and *E. coli* (inhibition halo measured 20 mm) while no activity was observed against the yeast *C. albicans* (Figure 11).

These data supported the choice of FG90 in the final formulation both as stabilizing agent of the formulation and as antimicrobial agent that could support the activity of the extracts.

The final O/W emulgel composition, using 30/70 *w*/*w* O/W ratio was the following:

- oil phase (O): L.E. 22.0 g, cetostearyl alcohol 6.0 g, cetomacrogol 1000 2.0 g
- water phase (W): FG90 1% wt. solution 69.0 g, D.E. 1.0 g.

### 3.4. Emulgel Characterization

#### 3.4.1. Organoleptic Properties and Stability

**Figure 12:**
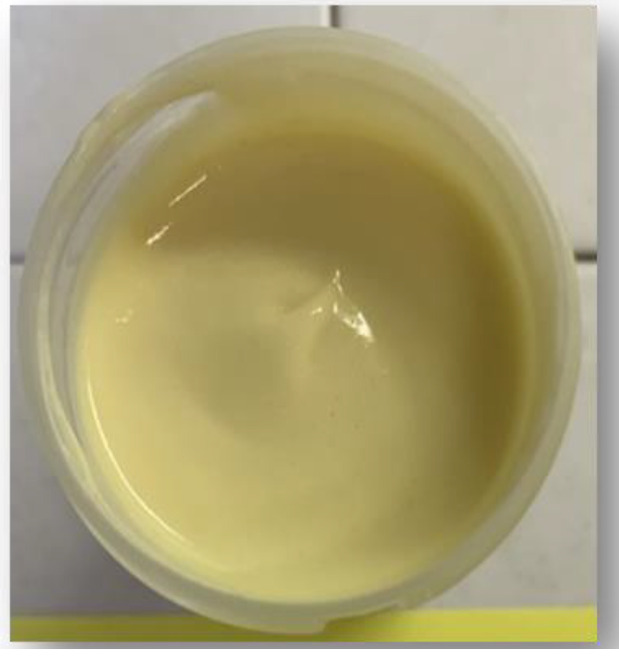
Emulgel loaded with the two flaxseed extracts.

A preliminary characterization of the prepared emulgel consisted in the evaluation of the organoleptic properties. The emulgel has been tried by five people on the hand skin, and a rating has been given. The emulgel for all five people is pleasant, fresh, non-greasy, has good spreadability and good consistency. All people said that the skin is softener and smoother after emulgel application. Moreover, the emulgel appears to be uniform, not shiny (index of a good emulsion) and light-yellow colored (Figure 12).

#### 3.4.2. Droplet Size Measurement

**Figure 13:**
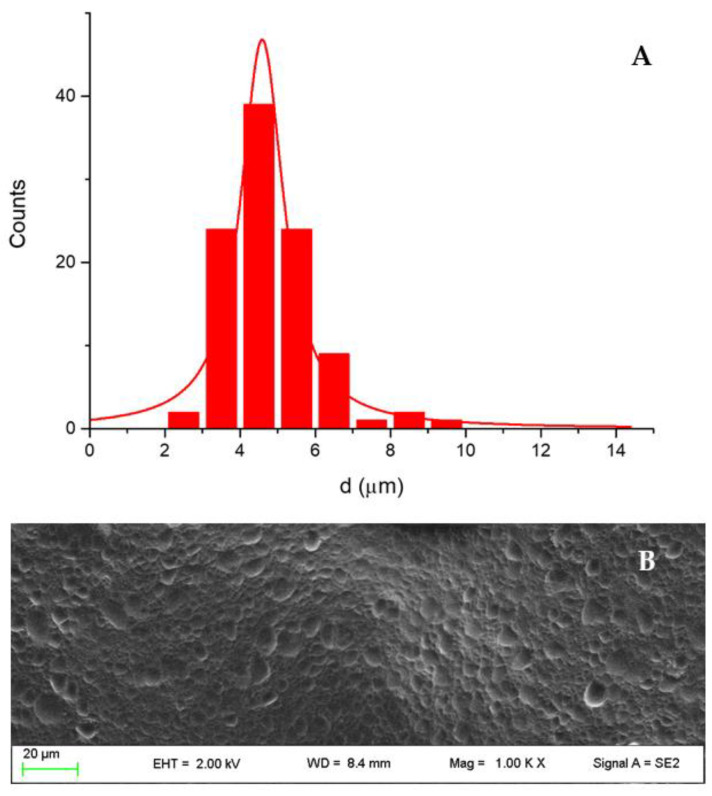
(**A**) Dimensional distribution of the internal oil phase droplets; (**B**) SEM micrograph of the emulgel.

The dimensions of the oil internal phase droplets were acquired from SEM micrographs and statistically elaborated by ImageJ software using SEM images. The mean diameter was 5.0 ± 1.0 µm (Figure 13).

#### 3.4.3. Antimicrobial Activity

In order to evaluate emulgel antimicrobial activity, a further study was planned. The assay was performed using the modified agar diffusion method (Section 2.3).

The emulgel (~300 mg) was seeded in a hole previously made in the center of Petri dishes containing agar with the same strains tested for D.E., L.E. and FG90 (Table 1).

**Figure 14:**
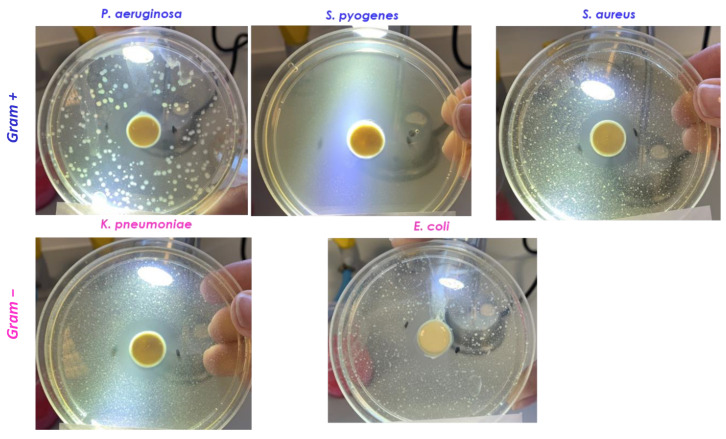
Inhibition halos measured for the emulgel.

The obtained results were very surprising as different from those obtained from the raw materials (Table 4). In fact, the emulgel was particularly active against the Gram+ bacteria *S. pyogenes* and *S. aureus* and against the Gram− bacteria *P. aeruginosa*, *K. pneumoniae* and *E. coli* as testified by the inhibition halos measured (Table 4, Figure 14). These results could be supported by the results obtained from other authors which observed that the combination of low molecular weight chitosan with conventional antibiotics as gentamicin, erythromycin, vancomycin, ciprofloxacin promotes an increase of their antibacterial activity by decreasing their MIC values [25]. Ali et al. demonstrated the ability of chitosan to decrease the minimum inhibitory concentration (MIC) of some antimicrobial drugs against *P. aeruginosa*; e.g., sulfamethoxazole MIC decreased until 60 times [24].

Based on these findings, further studies were planned with the aim to measure MIC and MBC values against *S. pyogenes* (the strain particularly sensitive to the emulgel) of the emulgel, as well as the raw materials alone (FG90, D.E. and L.E.).

Before to comment the obtained results (Table 5), it is important to take into account the following considerations. In 5.20 mg of emulgel (MIC value of the emulgel 5.20 mg/mL, Table 5) are contained 0.052 mg/mL of D.E., 1.14 mg of L.E. and 0.036 mg of FG90, calculated taking into account the emulgel recipe reported in Section 3.3.

From these data emerges that combining D.E., L.E. and FG90 in the emulgel the MIC value is reduced compared to the raw materials alone (Table 5).

These findings are very important as the synergism allows one to obtain better antimicrobial effect (Table 4), also in strains not sensitive to the extracts alone (Table 3), using low D.E. amounts. This allows to avoid the use of cytotoxic concentrations for both RAW 264.7 cells (cytotoxic concentration: 0.625 mg/mL) and HaCaT cells (0.8 mg/mL) and to obtain both antimicrobial and anti-inflammatory activities (the inhibition of pro-inflammatory cytokines release was observed at the concentration of 0.038 mg/mL).

#### 3.4.4. Rheological Characterization

**Figure 15:**
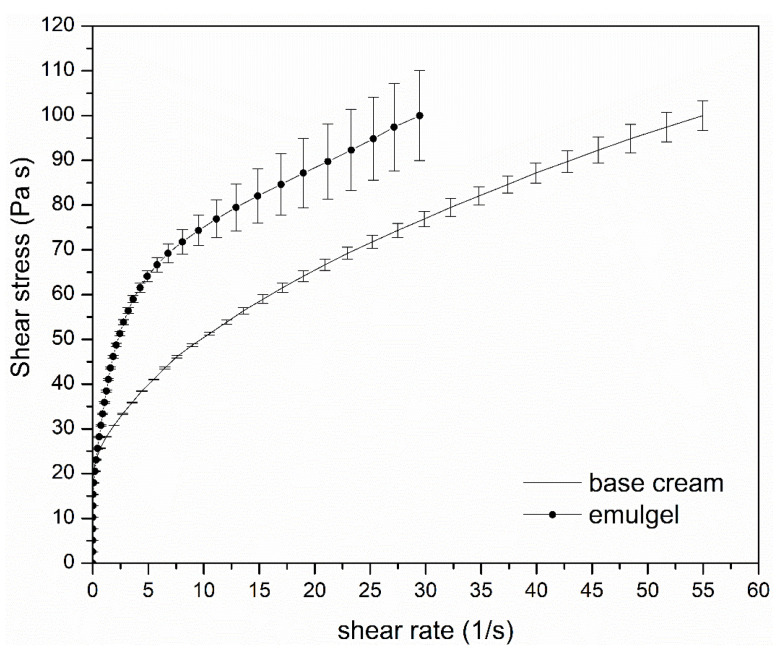
Rheogram of the base cream and of the developed emulgel.

The developed emulgel and the base cream, prepared according to F.U. XII Ed. recipe [23], prepared without extracts and used as control, were submitted to rheological measurements in order to evaluate their viscosity at 32 °C, temperature of the application site. The obtained results (Figure 15) show a pseudoplastic behavior for both the formulations and the developed emulgel, as expected, shows a higher viscosity in comparison to the base cream, testified by the shear rate measured. This is mainly due to the viscosity of the external gelled water phase, responsible for the increased consistency of the formulation resulting in the improved stabilization of the internal oil phase. Despite the increased consistency, the emulgel demonstrated to flow at very low shear stress values (<20 Pa·s), meaning that it can be easily applied to damaged skin by light massage and without pain.

## 4. Conclusions

Medicinal products or medical devices based on flaxseed extracts are not available despite the extensive literature data documenting flaxseed activities.

A dry extract (D.E.) and liquid extract (L.E.) were prepared starting from flaxseed flour. These products were very interesting in wound treatment, as both showed antioxidant activity and D.E. demonstrated to inhibit the production of the pro-inflammatory cytokines NO and IL-6.

D.E. and L.E. also showed antimicrobial properties (D.E. against *S. pyogenes* and *S. aureus*; L.E. against *S. pyogenes*). However, the concentrations necessary to obtain this activity are cytotoxic for keratinocytes and macrophages. In order to exploit both the anti-inflammatory and antimicrobial activities, a formulation was developed combining the two extracts. An O/W emulgel was prepared containing L.E. in the internal oil phase (O) and D.E. in the external water phase (W) in which FG90 chitosan (low molecular weight and deacetylated 99.97%) was introduced as a viscosizing agent to stabilize the emulsion. The use of this excipient allowed one to obtain a formulation with important antimicrobial activity, compared to the extracts alone, toward *S. aureus*, *S. pyogenes*, *E. coli*, *P. aeruginosa,* and *K. pneumoniae* strains responsible for diabetic foot ulcer infections and are reported in the WHO list of antibiotic-resistant pathogens [32,33]. Moreover, it was demonstrated that FG90 chitosan can work synergistically with the extracts decreasing their MIC values (compared to that measured for the extracts alone) within the concentrations range safe for macrophages and keratinocytes. Thus, the developed formulation showing antioxidant, anti-inflammatory, and antimicrobial activities could represent a valuable approach for the local treatment of diabetic foot ulcers. Future studies will be necessary to evaluate the in vivo performances of the formulation.

## Figures and Tables

**Table 1:** Strains tested and growth conditions.

Strain	Growth Conditions
**Gram + bacteria**	
*Staphylococcus epidermidis* WDCM 00036	37 °C for 24 ± 2 h
*Enterococcus faecalis* WDCM 00087	37 °C for 24 ± 2 h
*Bacillus subtilis* WDCM 00003	30 °C for 24 ± 2 h
*Staphylococcus aureus* WDCM 00034	37 °C for 24 ± 2 h
*Streptococcus pyogenes* ATCC 19615	37 °C for 24–48 h
**Gram − bacteria**	
*Pseudomonas aeruginosa* WDCM 00025	25 °C for 24–48 h
*Klebsiella pneumoniae* WDCM 00097	37 °C for 24 ± 2 h
*Proteus mirabilis* WDCM 00023	37 °C for 24 ± 2 h
*Escherichia coli* WDCM 00013	37 °C for 24 ± 2 h
**Yeast**	
*Candida* CM 00054 *albicans* WD	25 °C for 24–72 h

**Table 2:** Total phenolic content and antioxidant activity for D.E. and L.E. samples.

Extract	TPC (Mean ± SD) mg GAE/g Dry Flaxseed Flour	FRAP (Mean ± SD) µmol Fe^2+^/g Dry Flaxseed Flour	ABTS (Mean ± SD) mg TE/g Dry Flaxseed Flour
D.E.	1.94 ± 0.09	15.73 ± 3.10	5.25 ± 0.35
L.E.	1.62 ± 0.01	11.69 ± 0.21	0.62 ± 0.04

**Table 3:** Inhibition halos measured for D.E. and L.E. and for the commercial flaxseeds dry extract and oil.

Strains	D.E. 100 mg/mL (mm)	D.E. 150 mg/mL (mm)	Marketed D.E. 100 mg/mL (mm)	Marketed D.E. 150 mg/mL (mm)	L.E. (mm)	Marketed Flaxseed Oil (mm)
**Gram +**						
*S. epidermidis*	-	-	-	16	-	-
*E. faecalis*	-	-	-	-	-	-
*B. subtilis*	-	-	-	-	-	-
*S. aureus*	-	18	-	15	-	-
*S. pyogenes*	20	20	19	22	20	-
**Gram −**						
*P. aeruginosa*	-	-	-	-	-	-
*K. pneumoniae*	-	-	-	-	-	-
*P. mirabilis*	-	-	-	-	-	-
*E. coli*	-	-	-	-	-	-
**Yeast**						
*C. albicans*	-	-	-	-	-	-

-: no halo observed.

**Table 4:** Comparison of the inhibition halos obtained from the raw materials and the emulgel.

Strain	D.E. (150 mg/mL)	L.E. (0.87 mg/mL)	FG90 (1% wt.)	Emulgel	Base Cream (Control)
*S. aureus* WDCM 00034	18	-	-	24	-
*S. pyogenes* ATCC 19615	20	20	25	36	-
*P. aeruginosa* WDCM 00025	-	-	20	31	-
*K. pneumonia* WDCM 00097	-	-	23	27	-
*E. coli* WDCM 00013	-	-	20	26	-

**Table 5:** MIC and MBC against *S. pyogenes* values measured for the raw materials and the emulgel.

Sample	MIC (mg/mL)	MBC (mg/mL)
Ciprofloxacin (control)	1 μg/mL	1 μg/mL
D.E.	0.59	1.17
L.E.	0.22	0.44
FG90	0.30	0.30
Emulgel	5.20	5.20

## Data Availability

Not applicable.

## References

[1] Jhala A.J., Hall L.M. (2010). Flax (*Linum usitatissimum* L.): Current Uses and Future Applications. Aust. J. Basic Appl. Sci..

[2] Parikh M., Netticadan T., Pierce G.N. (2018). Flaxseed: Its bioactive components and their cardiovascular benefits. Am. J. Physiol. Heart Circ. Physiol..

[3] Shim Y.Y., Gui B., Arnison P.G., Wang Y., Reaney M.J.T. (2014). Flaxseed (*Linum usitatissimum* L.) bioactive compounds and peptide nomenclature: A review. Trends Food Sci. Technol..

[4] Wang H., Wang J., Qiu C., Ye Y., Guo X., Chen G., Li T., Wang Y., Fu X., Liu R.H. (2017). Comparison of phytochemical profiles and health benefits in fiber and oil flaxseeds (*Linum usitatissimum* L.). Food Chem..

[5] Kitts D.D., Yuan Y.V., Wijewickreme A.N., Thompson L.U. (1999). Antioxidant activity of the flaxseed lignin secoisolariciresinoldiglycoside and its mammalian lignan metabolites enterodiol and enterolactone. Mol. Cell. Biochem..

[6] Marand M.A., Amjadi M.S., Marand M.A., Roufegarinejad L., Mahdi Jafari S. (2020). Fortification of yogurt with flaxseed powder and evaluation of its fatty acid profile, physicochemical, antioxidant, and sensory properties. Powder Technol..

[7] Kyselka J., Rabiej D., Dragoun M., Kreps F., Burčová Z., Němečková I., Smolová J., Bjelková M., Szydłowska-Czerniak A., Schmidt Š. (2017). Antioxidant and antimicrobial activity of linseed lignans and phenolic acids. Eur. Food Res. Technol..

[8] Kaithwas G., Mukerjee A., Kumar P., Majumdar D.K. (2011). *Linum usitatissimum* (linseed/flaxseed) fixed oil: Antimicrobial activity and efficacy in bovine mastitis. Inflammopharmacology.

[9] Kajla P., Sharma A., Sood D.R. (2015). Flaxseed, a potential functional food source. Int. J. Food Sci. Technol..

[10] Rafiee S., Nekouyian N., Hosseini S., Sarabandi F., Chavoshi-Nejad M., Mohsenikia M., Yadollah-Damavandi S., Seifaee A., Jangholi E., Eghtedari D. (2017). Effect of Topical *Linum usitatissimum* on Full Thickness Excisional Skin Wounds. Trauma Mon..

[11] Pagano C., Marinozzi M., Baiocchi C., Beccari T., Calarco P., Ceccarini M.R., Chielli M., Orabona C., Orecchini E., Ortenzi R. (2020). Bioadhesive Polymeric Films Based on Red Onion Skins Extract for Wound Treatment: An Innovative and Eco-Friendly Formulation. Molecules.

[12] Pagano C., Perioli L., Baiocchi C., Bartoccini A., Beccari T., Blasi F., Calarco P., Ceccarini M.R., Cossignani L., di Michele A. (2020). Preparation and characterization of polymeric microparticles loaded with Moringa oleifera leaf extract for exuding wound treatment. Int. J. Pharm..

[13] Pagano C., Perioli L., Blasi F., Bastianini M., Chiesi C., Cossignani L. (2017). Optimisation of phenol extraction from wine using layered double hydroxides and technological evaluation of the bioactive-rich powder. Int. J. Food Sci. Technol..

[14] Pollini L., Tringaniello C., Ianni F., Blasi F., Manes J., Cossignani L. (2020). Impact of ultrasound extraction parameters on the antioxidant properties of Moringa oleifera leaves. Antioxidants.

[15] Pollini L., Rocchi R., Cossignani L., Mañes J., Compagnone D., Blasi F. (2019). Phenol profiling and nutraceutical potential of Lycium spp. leaf extracts obtained with ultrasound and microwave assisted techniques. Antioxidants.

[16] Balouiri M., Sadiki M., Ibnsouda S.K. (2016). Methods for in vitro evaluating antimicrobial activity: A review. J. Pharm Anal..

[17] Ceccarini M.R., Vannini S., Cataldi S., Moretti M., Villarini M., Fioretti B., Albi E., Beccari T., Codini M. (2016). In Vitro Protective Effects of *Lycium barbarum* Berries Cultivated in Umbria (Italy) on Human Hepatocellular Carcinoma Cells. BioMed Res. Int..

[18] Pagano C., Perioli L., Latterini L., Nocchetti M., Ceccarini M.R., Marani M., Ramella D., Ricci M. (2019). Folic acid-layered double hydroxides hybrids in skin formulations: Technological, photochemical and in vitro cytotoxicity on human keratinocytes and fibroblasts. Appl. Clay Sci..

[19] Teh S.S., Bekhit A.E.D., Birch J. (2014). Antioxidative polyphenols from defatted oilseed cakes: Effect of solvents. Antioxidants.

[20] Deng Q., Yu X., Ma F., Xu J., Huang F., Huang Q., Sheng F. (2017). Comparative analysis of the in-vitro antioxidant activity and bioactive compounds of flaxseed in China according to variety and geographical origin. Int. J. Food Prop..

[21] Tawaha K., Alali F., Gharaibeh M., Mohammad M., El-Elimat T. (2007). Antioxidant activity and total phenolic content of selected Jordanian species. Food Chem..

[22] Hoel T., Casals J.B., Eng J. (1993). In vitro antimicrobial susceptibility testing of rapidly growing mycobacteria using the tablet diffusion method: Resistance pattern of Norwegian Mycobacterium fortuitum and Mycobacterium chelonae isolates. APMIS.

[23] Istituto Superiore di Sanità (2008). Macrogol Cetostearile Etere Crema e Unguento Base.

[24] Tin S., Sakharkar K.R., Lim C.S., Sakharkar M.K. (2009). Activity of Chitosans in combination with antibiotics in Pseudomonas aeruginosa. Int. J. Biol. Sci..

[25] Asli A., Brouillette E., Ster C., Ghinet M.G., Brzezinski R., Lacasse P., Jacques M., Malouin F. (2017). Antibiofilm and antibacterial effects of specific chitosan molecules on Staphylococcus aureus isolates associated with bovine mastitis. PLoS ONE.

[26] Ali A., Ahmed S. (2018). A review on chitosan and its nanocomposites in drug delivery. Int. J. Biol. Macromol..

[27] Perioli L., Ambrogi V., Pagano C., Scuota S., Rossi C. (2009). FG90 chitosan as a new polymer for metronidazole mucoadhesive tablets for vaginal administration. Int. J. Pharm..

[28] Filimon M.N., Popescu R., Sinitean A., Dumitrescu G. (2018). The Assessment of Chitosan Solutions Effects on Bacterial Strains. Rev. Chim..

[29] Matica M.A., Aachmann F.L., Tøndervik A., Sletta H., Ostafe V. (2019). Chitosan as a Wound Dressing Starting Material: Antimicrobial Properties and Mode of Action. Int. J. Mol. Sci..

[30] Goy R.C., de Britto D., Assis O.B.G. (2009). A review of the antimicrobial activity of chitosan. Polimeros.

[31] Friedman A.J., Phan J., Schairer D.O., Champer J., Qin M., Pirouz A., Blecher-Paz K., Oren A., Liu P.T., Modlin R.L. (2013). Antimicrobial and Anti-Inflammatory Activity of Chitosan–Alginate Nanoparticles: A Targeted Therapy for Cutaneous Pathogens. J. Investig. Dermatol..

[32] (2019). *No Time to Wait: Securing the Future from Drug-Resistant Infections*; Report to the secretary general of the United Nations. https://www.who.int/antimicrobial-resistance/interagency-coordination-group/final-report/en.

[33] WHO Publishes List of Bacteria for Which New Antibiotics Are Urgently Needed. Report OMS February 2017. https://www.who.int/news-room/detail/27-02-2017-who-publishes-list-of-bacteria-for-which-new-antibiotics-are-urgently-needed.

